# Application of a new coaxial bipolar electrode for the treatment of vertebral metastases: a pilot study in an ovine model

**DOI:** 10.3389/fmed.2025.1627296

**Published:** 2025-09-24

**Authors:** Francesca Salamanna, Matilde Tschon, Giuseppe Tedesco, Lucia Martini, Melania Maglio, Luca Cavazza, Noemi Dolciotti, Federico Rossano, Micaela Liberti, Simona Salati, Matteo Cadossi, Davide Maria Donati, Laura Campanacci, Gianluca Giavaresi, Alessandro Gasbarrini, Milena Fini

**Affiliations:** ^1^Surgical Sciences and Technologies, IRCCS Istituto Ortopedico Rizzoli, Bologna, Italy; ^2^Department of Spine Surgery, IRCCS Istituto Ortopedico Rizzoli, Rizzoli, Bologna, Italy; ^3^Department of Information Engineering, Electronics and Telecommunications (DIET), Sapienza University of Rome, Rome, Italy; ^4^Italian Institute of Technology, Sapienza University of Rome and CLN^2^S@Sapienza, Rome, Italy; ^5^Medical Division, IGEA, Carpi, Italy; ^6^3rd Orthopaedic and Traumatologic Clinic Prevalently Oncologic, IRCCS Istituto Ortopedico Rizzoli, Bologna, Italy; ^7^Department of Biomedical and Neuromotor Sciences, Alma Mater Studiorum, University of Bologna, Bologna, Italy; ^8^Scientific Direction, IRCCS Istituto Ortopedico Rizzoli, Bologna, Italy

**Keywords:** electroporation, coaxial bipolar electrode, spine metastases, ovine model, computational simulation

## Abstract

**Introduction:**

Spinal metastases account for approximately 90% of masses detected through spinal imaging. Therefore, it is imperative to advance therapies. Electroporation modifies the permeability of cancer cell membranes using electric energy, increasing the local uptake of chemotherapeutics and promoting local tumor control. The aim of this study was to evaluate the safety of delivering electrical pulses that induce tissue ablation using novel coaxial bipolar electrodes in healthy bone and clinically relevant structures in an ovine model.

**Methods:**

Electroporation was performed on sheep vertebral bodies (L2-L4) applying an electric field intensity sufficient to deliver at least 3,500 J/Kg, absorbed energy previously shown to be effective in ablating bone tissue. The study also examined the impact on surrounding sensitive structures, such as peripheral nerves and the spinal cord. Effectiveness and safety assessment was performed by clinical evaluation, histological analysis and numerical simulation.

**Results:**

The results showed that the ablation induced by electroporation was clearly visible 7 days after treatment. This was confirmed histologically by the absence of osteoblasts, the complete inhibition of bone apposition, the presence of pyknotic osteocytes and the empty lacunae. The absence of tetracycline fluorescence further confirmed the absence of bone tissue growth in the ablated area. Histomorphometric analysis showed a significant difference (*p <* 0.0005) in the ablated area between the L2 vertebral body (where the electric field was applied with a single bipolar electrode; ablation area: 99.56 ± 18.00 mm^2^) and L3 and L4 vertebrae (where the electric field was applied by 2 bipolar electrodes; ablation area: 238.97 ± 81.44 mm^2^). Computational modeling showed that the estimated ablation volume was 0.43 cm^3^ in L2 and 3.45 cm^3^ in L4. Furthermore, no deficits following the application of the electrical pulses were observed in spinal nerves and spinal cord.

**Discussion:**

In agreement with these findings, temperature estimation based on computational simulation showed negligible increase in the spinal cord at the level of treated vertebra. Utilizing coaxial bipolar electrodes within the vertebral body through a transpedicular approach could offer a safe and minimally invasive procedure to treat spinal tumors and metastases, regardless of lesion size, while safeguarding critical neural structures.

## Background

Metastases frequently affect the spine, especially from primary carcinomas of the breast, prostate, thyroid, and lung (1). Bone metastases can cause severe pain and have a detrimental effect on quality of life, resulting in an immeasurable social and economic burden (1–2). As systemic pathologies, they require multidisciplinary therapeutic approaches. The treatment of anatomical sites such as the vertebrae adds to the complexity of managing this condition (3–4), mainly due to the presence of the spinal cord and nerves, which makes it particularly challenging (5).

In addition, spinal metastases display a wide range of characteristics and behaviors due to the diverse histotypes and modes of spread of the primary tumor (6). Currently, there are several therapeutic approaches and strategies available for treating metastases in the vertebral region. However, surgery may not always be feasible due to the location, and the patient’s clinical condition (7–9). Therefore, there is a growing interest in new minimally invasive treatment modalities, such as radiation therapy, cryotherapy, and percutaneous radiofrequency ablation (10). However, patients often report inadequate benefits from these treatments, which can reduce the mechanical strength of the bone and require additional procedures to stabilize the treated bone segments.

Electroporation (EP) is a physical technique that uses microsecond-length electric pulses to temporarily alter the cell membrane permeability without compromising cell viability, a process known as reversible electroporation (REP) (11). Reversible electroporation of the cell membrane is utilized for gene delivery (electrogene transfer, EGT) and for the delivery of cytotoxic drugs (electrochemotherapy, ECT) (12–14). However, the application of a high number (80–120) of electrical pulses may directly induce cell death, resulting in irreversible electroporation (IRE) (11).

ECT has been in clinical use since 2006, building upon extensive preclinical studies that have defined the parameters for effective tumor treatment and elucidated the mechanisms of action (15–17). The primary advantage of ECT is the ability to locally enhance the cytotoxic effect of low permeant chemotherapeutic drugs by increasing the permeability of cell membranes, while containing systemic side effects (12–14). Furthermore, the application of an electric field induces transient vasoconstriction, resulting in hypo-perfusion of the treated area, which is particularly effective in tumor tissues with disorganized and immature vascularization, limiting drug washout (12–14). The procedure necessitates shorter treatment times compared to thermoablation and cryoablation techniques and it also preserves the regenerative capacity of local healthy bone tissue and does not compromise the mechanical competence of the mineralized tissue (18).

In recent years, ECT has been used as minimally invasive treatment for primary and metastatic tumor lesions in various anatomical sites (19–23). At present, there are two main types of electrodes that are used for the application of the electric fields: plate electrodes and needle electrodes (24). Plate electrodes are mainly used for treating superficial accessible lesions (24). On the other hand, needle electrodes can be used for deep seeded tumor nodules treatments.

The feasibility of the EP procedure on the spine was previously evaluated by our research group in a sheep model, with the application of 4 electrode needles connected in consecutive pairs at the level of the lumbar vertebrae L3-L4 (25). The study found that despite the impact of the applied electric field on tissues between electrodes, the structural integrity of bone and extracellular matrix were maintained. Transient effects, such as interstitial edema and vacuolization of spinal nerves and the spinal cord, were observed but did not cause permanent functional deficits (25). This preclinical study led to the clinical application of the method for treating vertebral metastases (26). Specifically, Gasbarrini et al. treated vertebral metastasis from melanoma with ECT for the first time (26). The procedure involved the insertion of needle electrodes directly into the vertebral bodies after partial laminectomy and intravenous injection of bleomycin (26). Later, Deschamps and colleagues reported the results of ECT treatment in forty patients with radiotherapy-resistant metastatic epidural spinal cord compression (MESCC) (27), showing that ECT can rescue radiotherapy-resistant MESCC (28). Although these results are extremely encouraging, it has been deemed necessary to simplify the procedure by using specific needle designs and geometries. This will reduce the number of electrode needles applied, insertion times, and invasiveness of the procedure while also increasing accuracy.

For this reason, a new bipolar coaxial electrode has been developed: the bipolar electrode differs from the already available electrodes (29). The bipolar electrode has been developed to reduce the number of needles required for tumor ablation simplifying electrode placement, minimizing invasiveness, and saving time.

The location of a metastatic tissue in the vertebral body can be detected when its size is small and does not affect most of the vertebral body. In the case of single, small metastases, EP treatment can be performed by inserting a single bipolar electrode through the vertebral pedicle, making the treatment simple and minimally invasive. If the metastasis is localized in the center of the vertebral body and occupies a large part of it, the use of two bipolar electrodes inserted through the respective pedicles allows the electrical pulses to be activated and combined across the four poles, completely covering the surface of the vertebral body.

The aim of the study was to evaluate the effectiveness of bipolar coaxial electrodes, used individually or in pairs, in ablating vertebrae bone tissue, including the vertebral body and pedicles, and to quantify the extent of the ablation volume. In addition, this study assessed the safety of delivering electric pulses in a large animal model, with specific attention to nearby clinically relevant structures such as the spinal cord and peripheral nerves. Safety evaluation was based on both clinical observations and histological analyses.

## Methods

### Electroporation settings and electrode configuration

A custom-made pulse generator (IGEA S.p.A, Carpi, Italy) was used for *in vivo* EP. Application of electrical pulses to the vertebrae was achieved through vertebrae pedicles, in bipolar coaxial electrode the anode and the cathode components are on the same needle. The following electrode needles configuration has been used: diameter of the needle 2.1 mm, conductive pole P1 length 5 mm, insulating spacer length 5 mm, conductive pole P2 length 10 mm.

Two different treatment settings were applied with the bipolar electrode: one setting was applied when the bipolar electrode was used as single electrode, the other setting was applied when two bipolar electrodes were used in pairs. A schematic representation of the treatments settings is provided in the Supplementary Figure 1. Briefly, EP settings for single bipolar electrode: 8 bursts composed of 10 square-wave electrical pulses 100 μs long were delivered at a pulse frequency of 1 kHz. Tension applied between conductive poles was set at 800 V. EP settings for two bipolar electrodes used in pairs: 8 bursts composed of 10 square-wave electrical pulses 100 μs long at a pulse frequency of 1 kHz were delivered between each conductive pole. The sequences applied were as follows: 1–2: 800 V; 3–4: 800 V; 1–3: 1600 V; 2–4: 1200 V; 1–4: 1600 V; 2–3: 1600 V.

### Surgery

The *in vivo* study was performed in compliance with the European and Italian Laws (Legislative Decree No. 26/2014) on animal experimentation and in accordance with the environmental parameters specified by current European regulations and Recommendation 2007/526/EC, upon approval by the Animal Welfare Body of the IRCCS Istituto Ortopedico Rizzoli and authorization by the Ministry of Health (n° 257/2021-PR of April 9, 2021).

Six adult female sheep crossbred weighing 55 ± 5 kg (Tommaso Mioli, Budrio, Bologna, Italy) were used. The animals were housed in individual boxes with a grid floor at room temperature and controlled humidity. The animals were fed a standard maintenance diet (Mucedola, Settimo Milanese), clover and water *ad libitum*. After premedication with intramuscular administration of 44–50 mg/kg ketamina (Lobotor - Iniet Fl 10 mL 100 mg/ML, Acme Srl, Cavriago-RE) and 0.3 mg/kg xylazina (Rompum, Bayer S.p. A. Milan), general anesthesia was induced by intravenous bolus infusion of thiopental sodium 25 mg/mL, 0.25 mL/kg (Pentothal Sodium, MSD Animal Health Srl, Segrate MI). General anesthesia was maintained by assisted ventilation (Servo Ventilator, 900D; Siemens Italia, Milan, Italy) with the administration of O_2_/air 60%/40% (7 L/min) mixture with isoflurane 2–3% (Isoflurane-Vet, Merial Italia S.p.A., Milan).

Under general anesthesia and in a sterile environment, a posterior midline incision was made in the back, and the lumbar spine was exposed. Bipolar coaxial electrodes, equipped with a suitable tip for piercing the vertebral pedicle, were inserted via transpedicular approach into the body of the vertebra at the levels of the L2, L3, and L4 spinal segments. Subsequently, the EP protocol described above was applied at the level of each vertebral body. The treatment configuration involved the insertion of one coaxial bipolar electrode with electric field application in the L2 vertebral body, and the insertion of two coaxial bipolar electrodes with electric field application in pairs in the L3-L4 vertebral bodies.

In the L2 vertebral body, the two conductive poles of the electrode were connected in pairs. In the L3 and L4 vertebral bodies, the two conductive poles of the two electrodes were connected in consecutive pairs, and the same number of pulses was applied to each electrode couple in all the six possible combinations: four sides and two diagonals. Subsequently, two electrodes were inserted into the L5 vertebra, but no pulses were applied to study the effects of the perforation on the bony trabeculae.

During the postoperative period, analgesics (i.m. metamizole sodium, 50 mg/kg/day for 3 days, Farmolisina Ceva, Ceva Salute Animale S.p.A., Milano, Italy; i.m. ropivacaine hydrochloride 7.5 mg/mL in a single circling administration, Ropivacaina Cloridrato, S. A. L. F. S.p.A, Laboratorio Farmacologico, Cenate Sotto, Bergamo; transdermal patch of fentanyl 50 μg/72 h, Matrifen, Takeda SpA, Roma, Italy) were administered, and the sheep were housed in single boxes under the same environmental conditions. To label bone formation, oxytetracycline 30 mg/kg (Terramicina LA, Pfizer Italia Srl, Latina, Italy) was injected on the third day after surgery to assess the presence of bone tissue growth in the ablated area.

Seven days post-electroporation procedure, the animals undergo pharmacological euthanasia (Tanax® 20 mL/head) under deep general anesthesia (20 mg/kg ketamine and 0.6 mg/kg xylazine), followed by the removal of the treated (L2-L4) and untreated (L5) vertebral segments and other adjacent neurological structures: peripheral nerves and spinal cord. Upon removal, the treated and untreated vertebral segments were also imaged by X-ray (Nessey HF30-Raffaello-ACEM SpA).

### Histology and histomorphometry

Each vertebral body was sagittally bisected along the transverse (left–right) axis, resulting in two symmetrical halves. One half was not decalcified and was processed for resin embedding; the other half, together with vertebral pedicles, was decalcified in a 5% nitric-formic acid solution and embedded in paraffin.

The non-decalcified half of the vertebrae were first fixed in 4% paraformaldehyde, dehydrated in graded series of alcohols, and then embedded in polymethyl methacrylate (Merck, Schuchardt, Germany). Blocks were sectioned along a plane parallel to the vertical (cranio-caudal) long axis of the vertebra, passing through the sagittal midline (EXAKT GmbH & Co., Remscheid, Germany). Sections were then ground to a thickness of 30 ± 5.0 μm (Saphir apparatus 550, ATM GmbH, Germany) to evaluate tetracycline emission under fluorescence by a light microscope (*λ* = 410 nm; Olympus BX51).

The decalcified half of the vertebra and the vertebral pedicles were fixed in 10% formalin, together with the spinal cord and the spinal nerves. Subsequently, samples were extensively rinsed in distilled water, dehydrated in graded alcohol solutions, cleared in xylene and finally paraffin embedded. Sections (5 ± 1 μm) were taken by a semi-automated microtome (HM Leica microtome) and three slides for each sample were stained with hematoxylin and eosin (H&E). Histological images were taken with a digital pathology slide scanner (Aperio-Scanscope, Leica Biosystems, Germany). The maximum diameter of each ablated region was measured on the histological images acquired at 1x magnification using Aperio eSlide Manager software (Leica Biosystems). For consistency across all samples, including those treated with paired electrodes (L3 and L4), the diameter was defined as the maximum linear extent of the ablated region across its longest axis. Although these regions were not perfectly circular, the ablated area was estimated assuming a circular geometry using the formula: Area = *π* × (diameter/2)^2^.

### Statistical analysis

The statistical analysis was performed using the Microsoft Excel software. Data are reported as mean ± standard deviation (SD) at a significant level of *p <* 0.05. Ablated area data in the electroporated treated samples were analyzed statistically by using Student’s *t* test.

### Treatments numerical modeling

Numerical simulations were conducted to validate the effectiveness and the safety of the EP-mediated ablation procedure. The virtual sheep model was developed through the segmentation and 3D reconstruction of a whole-body CT scan of a sheep (30). The segmentation of all relevant anatomical structures – encompassing the spinal column, spinal cord, cerebrospinal fluid, and intervertebral discs – was performed using 3D Slicer. The resulting model was subsequently imported into COMSOL Multiphysics v.6.2, where the computational model for the treatments was established. 3D models of bipolar coaxial electrodes were designed in COMSOL by replicating the exact same geometry as those used during surgery. The electrodes were inserted within the vertebral body through the pedicles, as was done during the surgical procedure.

The electrical properties of the electrodes were incorporated into the simulation, while the dielectric properties of the biological tissues were assigned based on references (31–32) and the IT’IS Foundation database (33), as detailed in the Supplementary Table 1.

The tissue conductivity variation with electric field intensity was modeled using a smoothed Heaviside function, as described in (31).

The anatomical model was discretized using a multiresolution tetrahedral mesh in COMSOL, with element sizes ranging from approximately 0.02 mm to 2.5 mm, resulting in a total of about 3 million elements. This meshing approach provided a balance between anatomical detail and computational efficiency.

Simulations were run in the time domain using the “Electric Currents” physics interface in COMSOL, which solves the current conservation equation derived from Ohm’s Law. Further details on the governing equations can be found in the Supplementary Material, Section I.

To replicate the experimental stimulation protocol, each electrode pair was activated individually, using a separate simulation instance for each configuration. This approach mimicked the sequential pair-by-pair stimulation scheme applied during the *in vivo* procedures. The applied voltage waveform was modeled as a square pulse of 100 μs duration, consistent with the experimental conditions.mThe resulting contributions from different electrode pairs were then superimposed to mimic the experimentally observed biological effects.

The effectiveness of EP-mediated ablation was assessed by calculating the numerical absorbed dose (AD) in the target vertebral body and spinal cord, based on the absorbed dose equation described by Ibey BL et al. (34) and calculated as in Fini et al. (35). The AD threshold to achieve the EP-mediated ablation was set to 3,500 J/kg, which Fini et al. (35) proved to be effective in ablating bone tissue. Additionally, the temperature changes within the spinal cord were evaluated using an estimation of temperature rise derived from the Pennes’ bioheat equation (36), based on the computed specific absorption rate (SAR) values (see Supplementary Material, Section II). Specifically, thermal conduction, perfusion, and metabolic heat generation were neglected, assuming constant SAR over the exposure duration. The resulting temperature variation was estimated using the following Equation 1:


(1) $$\mathrm{\Delta T}\approx \frac{\mathrm{SAR}}{C}\cdot t_{\exp}$$

where *c* is the specific heat capacity of the tissue and t*_exp_* is the exposure time. This approximation provides a conservative estimate of the temperature increase and is suitable for preliminary safety evaluation.

## Results

### Clinical follow-up

One animal died 2 days post-operation due to a surgical complication associated with the procedure, specifically chronic bleeding caused by a muscle injury with a subserosal hemorrhagic collection in the right peritoneal region.

The remaining animals exhibited no changes in physiological habits and showed no alterations in gait, peripheral sensibility to hindquarters, or signs of pain with a fast recovery of quadrupedal station after surgery throughout the follow-up period. None of the animals had neurologic deficits.

**Figure 1:**
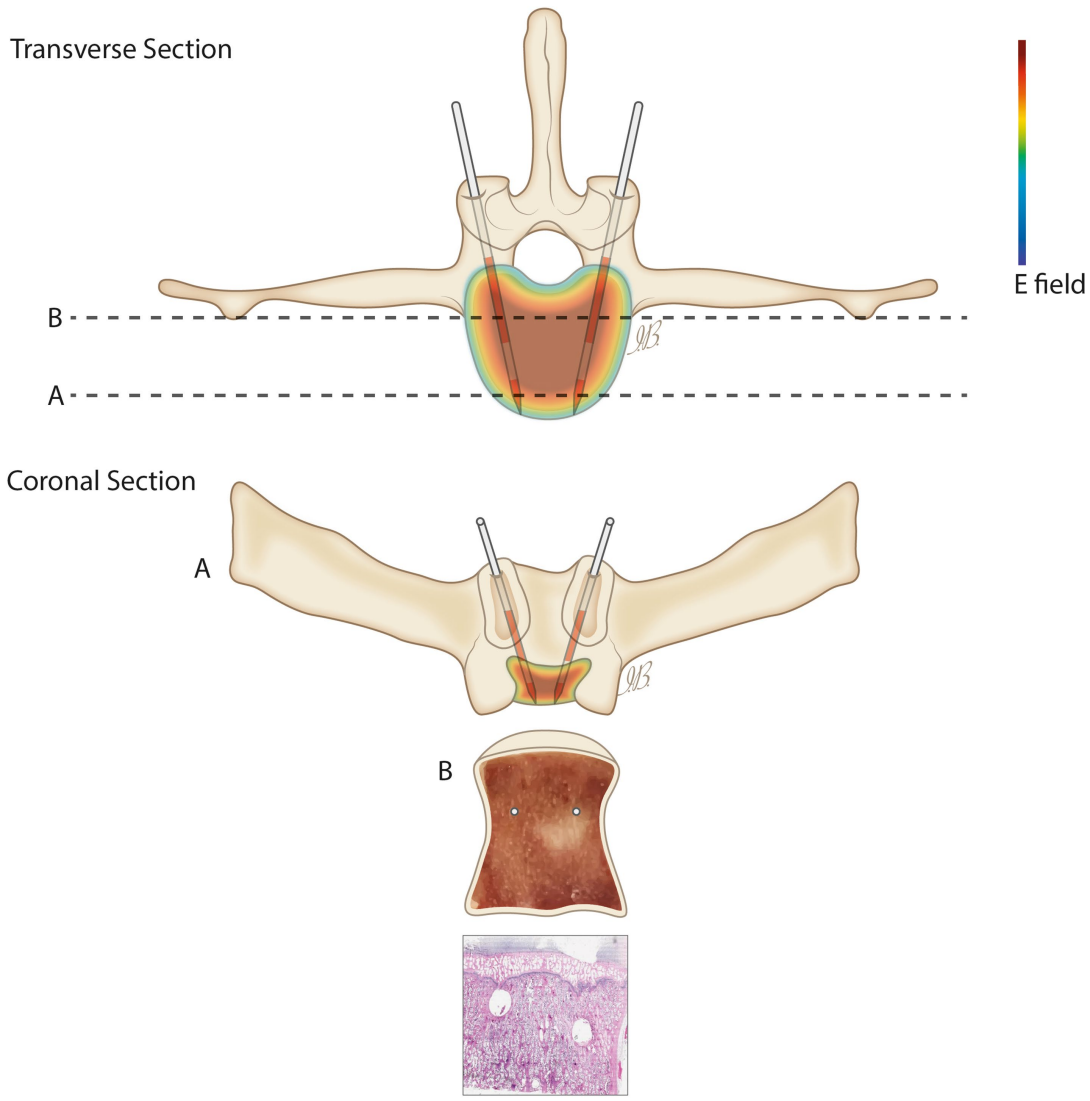
Schematic representation of the experimental setting with ideal electrode placement. Top panel: transverse schematic view of the vertebral body, showing the location of the electrodes and indicating two coronal section planes labeled A and B. Bottom panel: coronal views corresponding to section planes A and B as marked above. Panel **(A)** shows the anatomical view of the vertebral body along section A. Panel **(B)** includes the macroscopic image (top) and the corresponding H&E-stained histological section (bottom) obtained along section plane B.

Figure 1 shows a schematic representation of: the transverse view of the EP-mediated ablation applied to the sheep vertebral body and of the coronal view showing the *ex vivo* appearance of the explanted vertebral body (A), in which the electrodes were cut and left inserted as markers and of the obtained macroscopic and histological section, stained with Hematoxylin/Eosin, in which the ablation area was assessed (B).

### Histology and histomorphometry

#### Vertebral body

**Figure 2:**
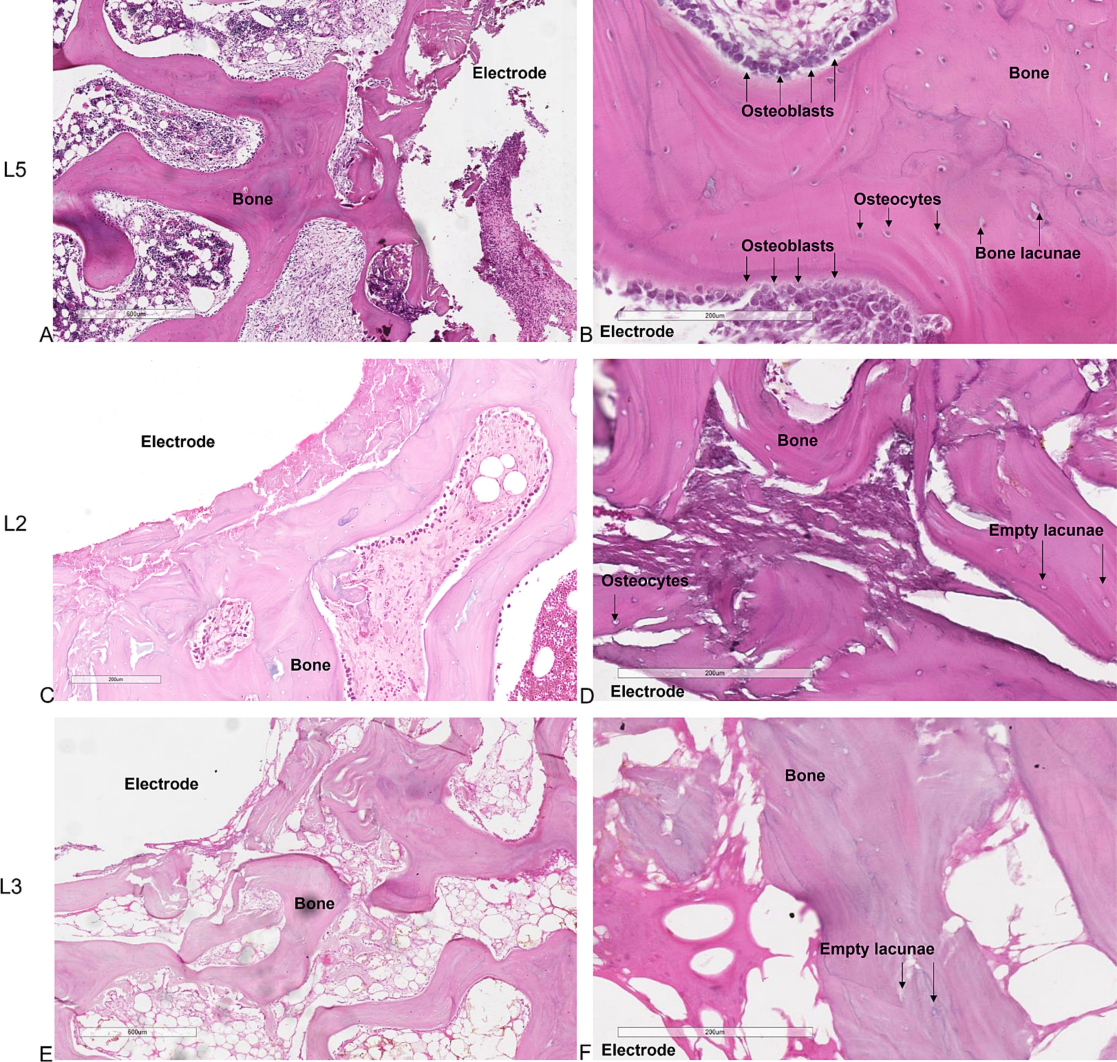
Histological sections of the vertebrae. **(A,B)** untreated L5 vertebral body. **(C,D)** application of the electric field with one bipolar electrode in L2 vertebral body. **(E,F)** application of the electric field with 2 bipolar electrodes in L3-L4 vertebral bodies. **(A,C,E)** × 4 magnification; **(B,D,F)** × 10 magnification, H&E staining.

Histological analysis of the untreated L5 vertebral body showed preexisting trabeculae among the electrodes entirely covered by osteoblasts, with evident evenly spread osteocytes (Figures 2A,B). These osteocytes were oriented with their longest axis in the direction of the lamellae contained within the bone lacunae, suggesting an active and systematic process of bone remodeling following the intervention. Conversely, in the vertebrae subjected to electroporation (L2, L3 and L4) we did not observe osteoblasts on trabeculae surface and osteocytes within the bone lacunae. In L2 the ablation was limited to the area surrounding the single bipolar electrode (Figures 2C,D). When 2 bipolar electrodes were inserted in the vertebral body (L3 and L4), ablation volume interested the whole vertebral body (Figures 2E,F). This indicates a significant and critical impact of electroporation-mediated ablation on the morphology and vitality of bone cells, potentially influencing the bone healing and regeneration process in the ablated regions.

**Figure 3:**
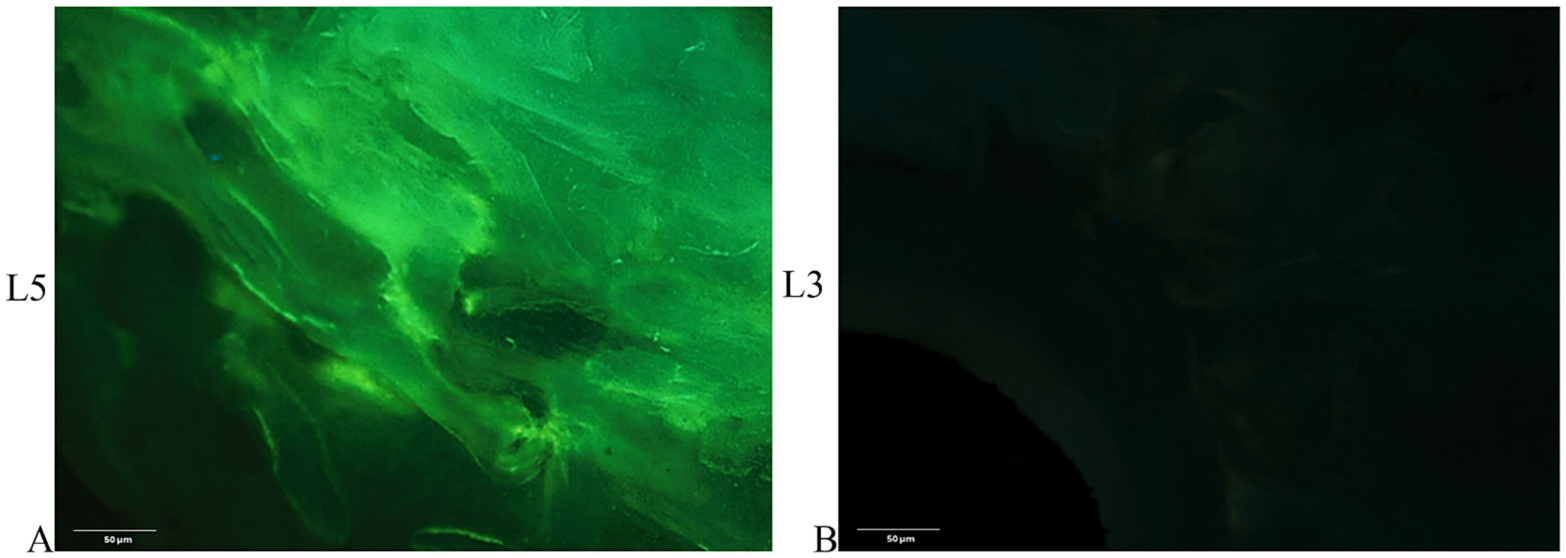
Tetracycline fluorescence emission for detecting new bone tissue growth. Fluorescence of non-decalcified bone tissue **(A)** around the electrodes in the untreated vertebra (L5) 7 days after electrode insertion (unstained section, ×20 magnification). **(B)** Absence of fluorescence in the L3 vertebral body 7 days after electroporation; (unstained section, ×20 magnification).

Tetracycline labeling in the untreated L5 vertebral body revealed the deposition of newly formed bone throughout the trabecular bone surrounding the electrodes, further underlining that the insertion procedure does not compromise bone tissue viability (Figure 3A). In contrast, in the electroporated vertebral bodies (L3-L4) tetracycline labeling was completely absent among the electrodes (Figure 3B).

**Figure 4:**
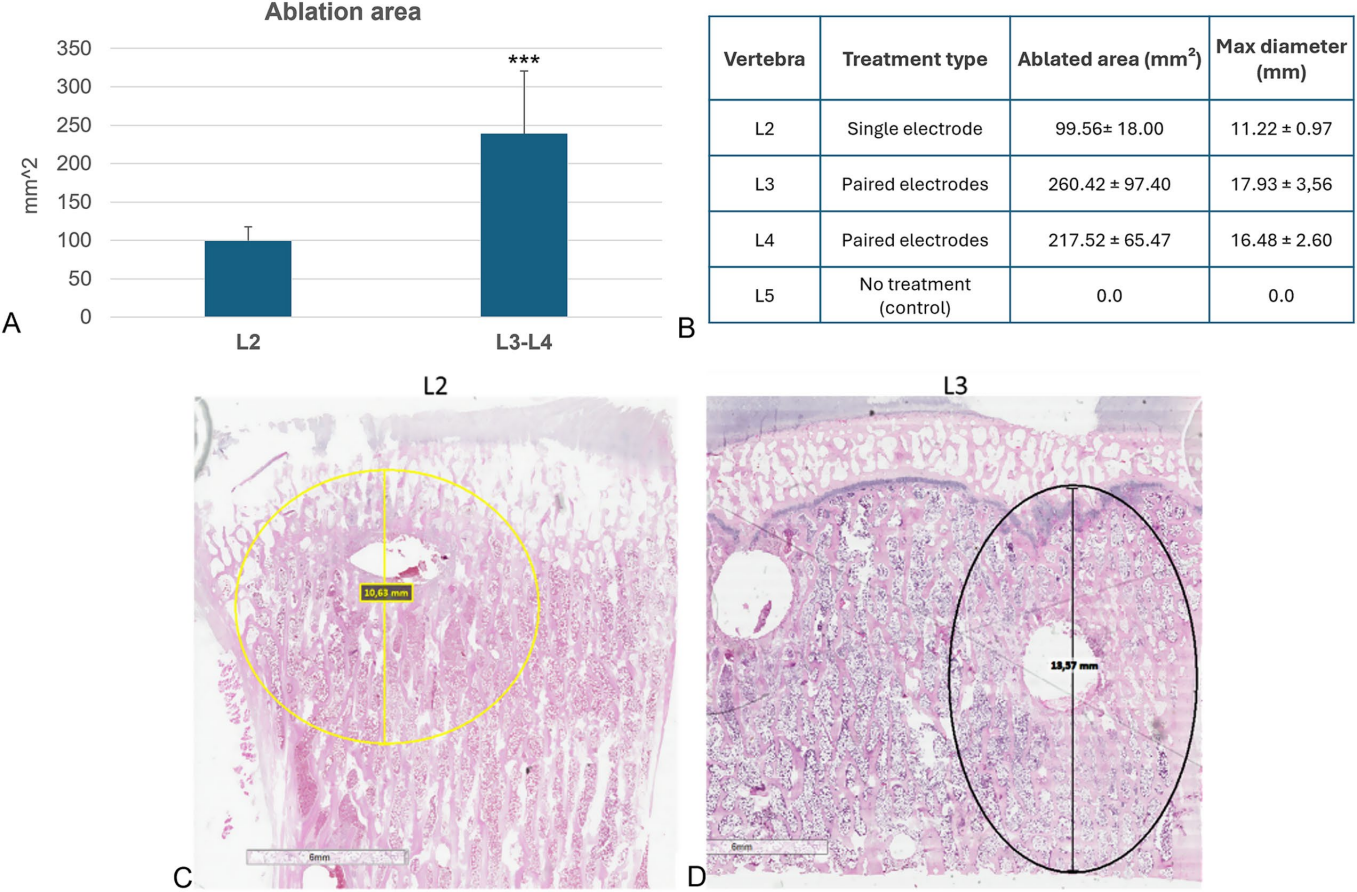
Histological and histomorphometric analyses of vertebral bodies. **(A)** Histomorphometric measurements of ablated area: L2 versus L3-L4 (Mean ± SD, *n* = 5): ***, *p* < 0.0005; **(B)** Table reporting individual measurements of the ablated area (mm^2^) and maximum ablation diameter (mm) for each vertebra analyzed. **(C)** Histological section of L2 vertebra: in yellow the ablated area of L2 vertebra (H&E staining); **(D)** Histological section of L3 vertebra: in black the ablated area of L3 vertebra (H&E staining).

Histomorphometric analysis showed that no ablated bone area was present in the L5 vertebrae, confirming that the surgical procedure did not affect cell viability. A significantly lower ablated area (*p <* 0.0005) was observed in the L2 vertebral body, where the electric field was applied to a single electrode, compared to the L3 and L4 vertebrae, where the electric field was applied to a pair of electrodes (Figure 4A). Detailed measurements of the ablated area and maximum diameter for each vertebra were reported in Figure 4B, further highlighting the difference between single and paired electrode treatments. These findings were further illustrated in the histological sections, where the ablated regions were clearly visible: in yellow in the L2 vertebra (Figure 4C) and in black in the L3 vertebra (Figure 4D), corresponding to the areas subjected to EP.

#### Vertebral pedicle

**Figure 5:**
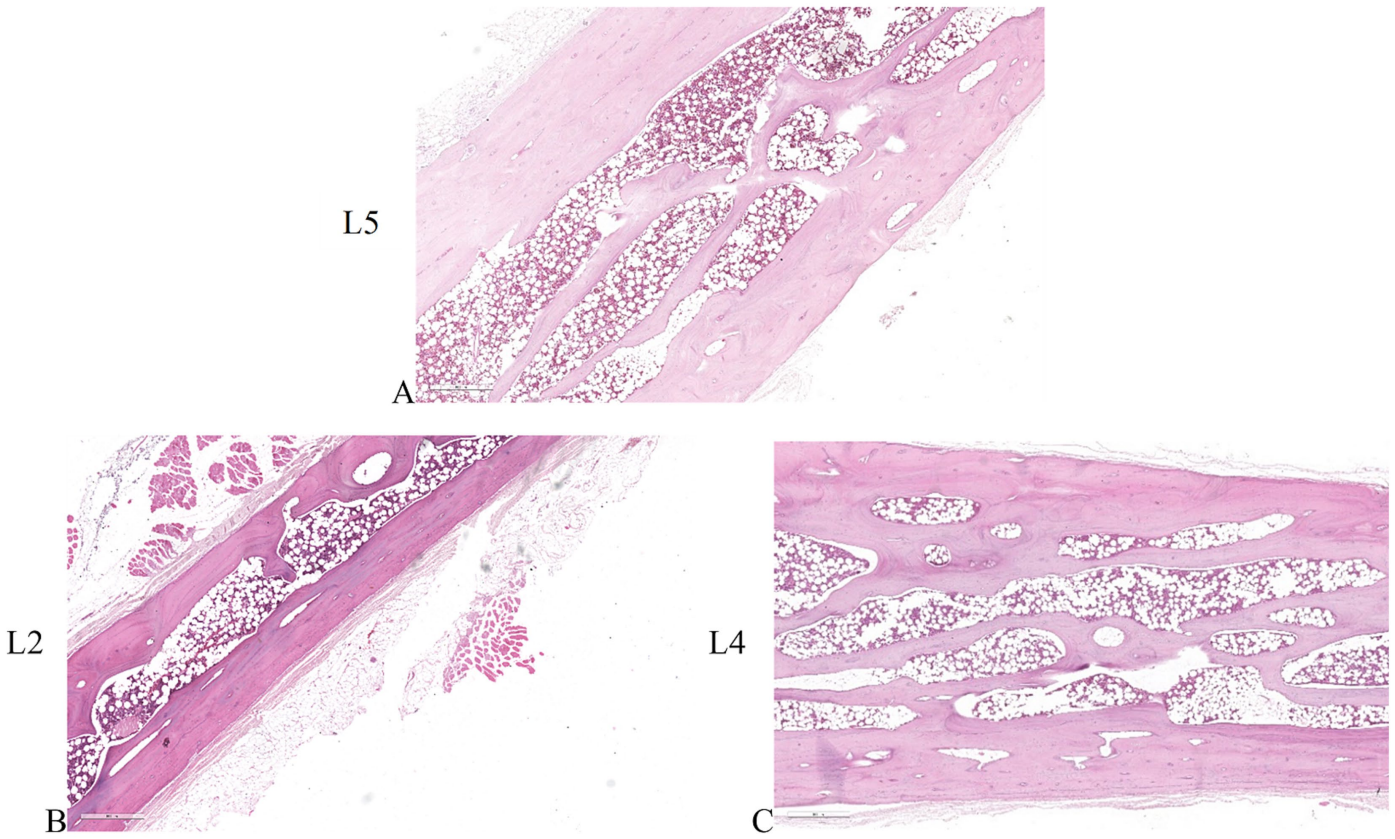
Histological sections of the vertebral pedicles. **(A)** vertebral pedicle of untreated L5 vertebral body. **(B)** vertebral pedicle of L2 vertebral body where the electric field was applied individually. **(C)** vertebral pedicle of L4 vertebral body where the electric field was applied as a pair. ×4 magnification, H&E staining.

Histological analysis of vertebral pedicles was conducted to evaluate any potential difference among untreated samples (Figure 5A) and those that underwent electroporation (Figures 5B,C). The examination revealed the same histological profile for all vertebral pedicles, characterized by the presence of bone trabeculae intricately woven with osteoblasts. Additionally, osteocytes were observed to be uniformly distributed within the bone lacunae, indicating a healthy and organized bone structure. Despite the application of electroporation, no discernible alterations in the microarchitecture or cellular composition of the vertebral pedicles were observed.

#### Spinal cord

**Figure 6:**
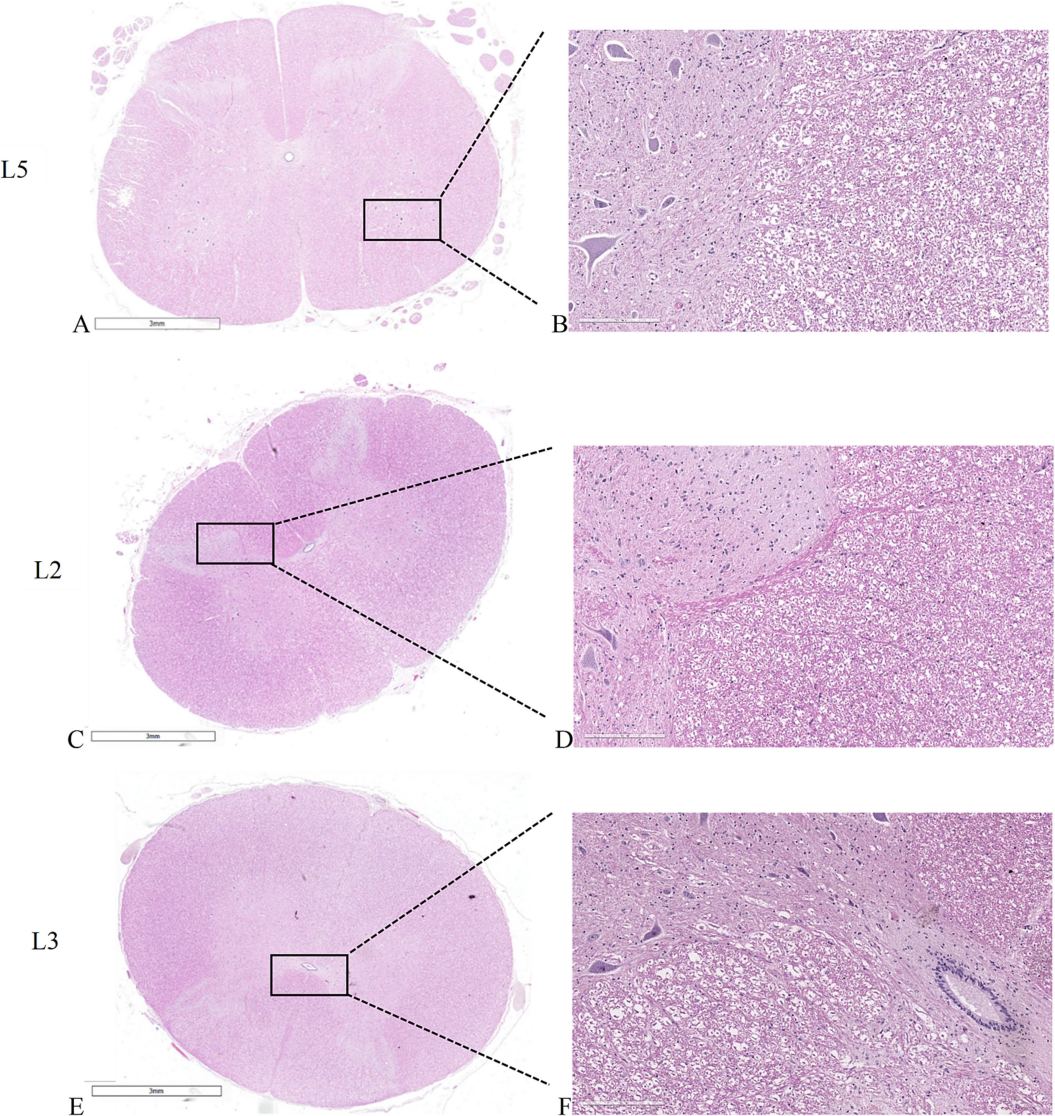
Histological sections of the spinal cords. **(A,C,E)** macro slide view. **(A,B)** untreated L5 spinal cord. **(C,D)** spinal cord adjacent to L2 vertebral body where the electric field was applied individually. **(E,F)** spinal cord adjacent to L3 vertebral body where the electric field was applied as a pair. **(A,C,E)** × 1 magnification; **(B,D,F)** × 20 magnification, H&E staining.

Figures 6A,B show the untreated spinal cord, with clearly distinguishable white matter, characterized by myelin sheaths surrounding cellular processes and a network of blood vessels, and gray matter, characterized by densely packed cells, fibers, and interconnected capillaries. Interestingly, when examining spinal cords adjacent to the electroporated bone, whether subjected to individual electric field application at the L2 vertebral body (Figures 6C,D) or simultaneous electric field application at the L3-L4 vertebral bodies (Figures 6E,F), both display consistent morphological and structural features like those observed in the untreated spinal cord. Furthermore, the vessels external to the spinal cord also exhibit normal structure and morphology, suggesting the absence of obstacles to normal blood flow and thus reducing the risk of ischemic conditions and other neurological complications related to vascular problems. All these aspects suggest that the application of the electric field did not alter the overall morphology and structure of the spinal cord.

#### Peripheral nerve

**Figure 7:**
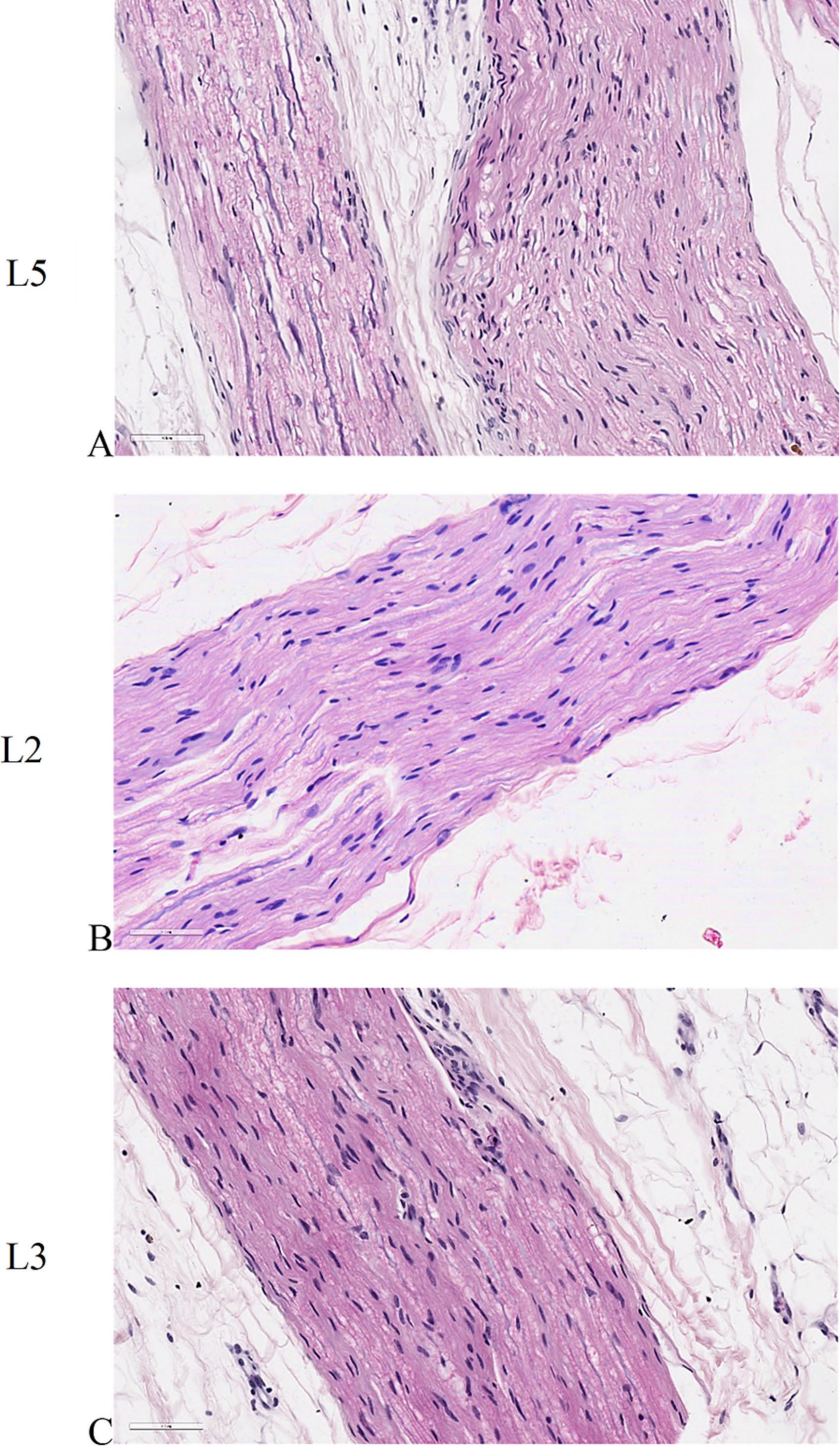
Histological sections of peripheral nerves. **(A)** untreated L5 peripheral nerve. **(B)** peripheral nerve adjacent to L2 vertebral body where the electric field was applied individually. **(C)** peripheral nerve adjacent to L3 vertebral body where the electric field was applied as a pair. ×40 magnification, H&E staining.

Longitudinal sections of peripheral nerve histology revealed a remarkable similarity between untreated specimens and those subjected to electroporation (Figures 7A–C). Notably, the individual nerve fibers exhibited a distinctive wavy appearance, indicative of their characteristic morphology. Within the nerve tissue, the prominent purple nuclei observed belonged to Schwann cells, which play a vital role in supporting and insulating nerve fibers. These Schwann cells were observed to intricately invest multiple nerve fibers with a neurilemma, forming a protective myelin sheath around each fiber. This structural arrangement is crucial for facilitating efficient nerve conduction and maintaining the integrity of the peripheral nervous system.

### Numerical assessment of treatments effectiveness and safety

**Figure 8:**
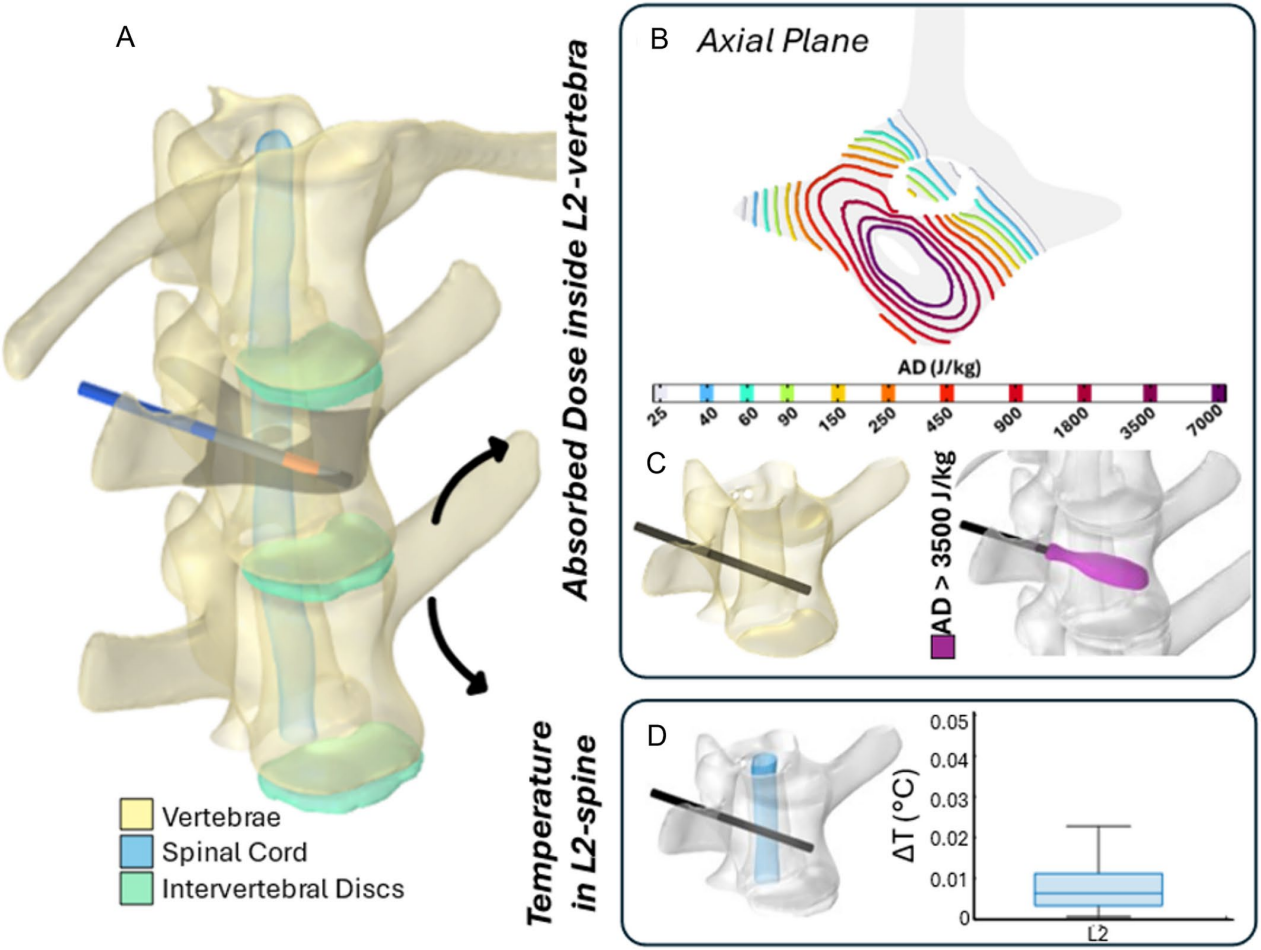
The 3D numerical model and results of simulated EP in L2 vertebra. Simulated treatment for EP-mediated ablation on L2. **(A)** Vertebral modeling and electrode positioning; **(B)** absorbed dose distribution (axial view); **(C)** resulting EP-mediated ablation volume (purple); **(D)** temperature variation within the spinal cord region highlighted in light blue.

**Figure 9:**
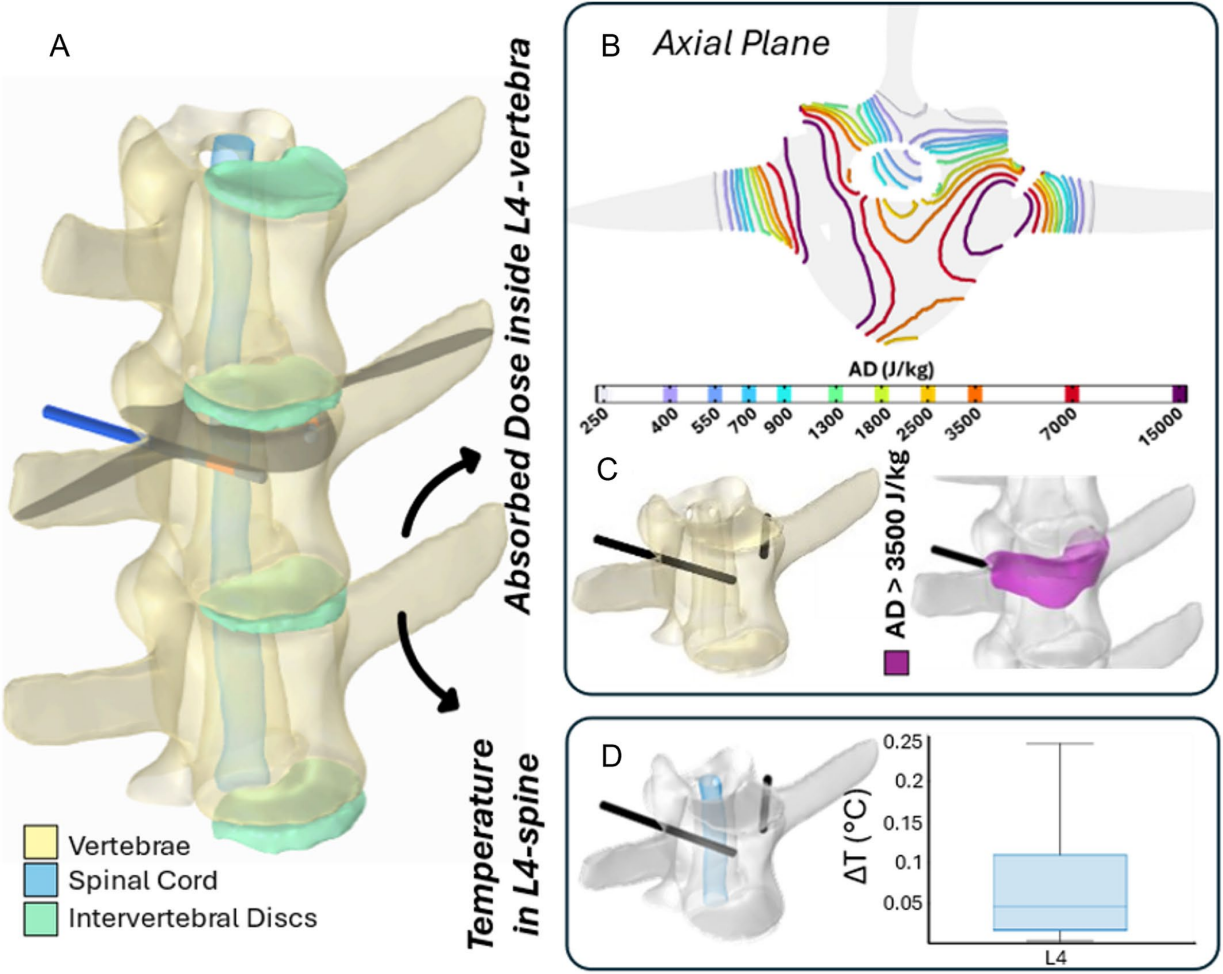
The 3D numerical model and the results of simulated EP in L4 vertebra. Simulated treatment for EP-mediated ablation on L4. **(A)** Vertebral modeling and electrode positioning; **(B)** absorbed dose distribution (axial view); **(C)** resulting EP-mediated ablation volume (purple); **(D)** temperature variation within the spinal cord region highlighted in light blue.

Figures 8, 9 illustrate the results of two representative cases for the treatment of the vertebra L2 using a single bipolar electrode and vertebra L4 using two bipolar electrodes, respectively.

Specifically, panels (A) depict the 3D anatomical model of the lumbar spine section – highlighting the vertebrae, spinal cord, and intervertebral discs – along with the electrodes positioning for each treatment. The black axial reference plane is placed across each vertebra to indicate the axial plane selected for the analysis of the AD (J/kg) distribution.

Figures 8B, 9B illustrate the distribution of the AD isolines within both the vertebral body and the spinal cord on the reference axial plane. In L2, the AD isolines exhibit a radial distribution originating from the active poles of the electrode. In L4, the radial pattern around each electrode is still observed, and the pairing of electrodes results in the AD coverage of the entire axial section of the vertebra, predicting a successful treatment in the upper L4 volume, as illustrated in Figure 9B. In both treatments, the spinal cord experiences low levels of AD, reaching maximum values up to 150 J/kg in L2 and 900 J/kg in L4.

Based on the AD threshold for tissue ablation of 3,500 J/kg (34), the estimated ablated volume within L2 and L4 vertebral bodies – depicted in purple in Figures 8C, 9C – is 0.43 cm^3^ and 3.45 cm^3^, respectively. In L2, the EP-mediated ablated region closely follows the shape of the electrode, resulting in a longitudinal distribution along the latter. In contrast, in L4, it covers a larger and more homogeneous portion of the vertebral body. The shape and size of the ablated volume depend on the specific electrode positioning and vertebrae anatomical features.

To further validate the safety of the procedure on the spinal cord, the temperature variation (ΔT) was estimated using Equation 1 and is presented in boxplots in Figures 8D, 9D. Specifically, the boxplots are based only on the portion of the spinal cord at the level of each treated vertebra [light blue region in panels (D)]. In both cases, the thermal effects on the spinal cord remain negligible, with a median ΔT below 0.01 °C for L2 and 0.05 °C for L4, reaching a maximum increase of 0.025 °C and 0.25 °C in L2 and L4, respectively.

## Discussion

Electroporation-based treatments, such as ECT and IRE ablation, have garnered considerable interest for their potential applications in medicine. When planning electroporation-based treatments, the main goal is to determine the best possible electrode position and applied voltage that will ensure the electroporation of the clinical target volume and cause minimal damage to the surrounding healthy tissue. IRE primarily induces cell death through apoptosis (37). However, other mechanisms like necrosis and immunogenic cell death can also occur. The impact of electric pulses varies depending on pulse characteristics, cell types, and treatment zones (37). While cells near the electrodes typically undergo necrosis, those at the periphery may survive through reversible electroporation (38). Combination therapies with cytotoxic drugs have been effective in tumor nodule ablation (38). IRE has shown promise in treating various tumors and maintaining vessel patency (38–41). However, there is limited data on its safety for spine-related structures. Previous studies have shown IRE’s efficacy in eliminating cells from vertebral body trabeculae in sheep without structural alterations (25). Several human spinal metastases cases have been treated with ECT so far: the first report dating back to 2015 (23–24). Since 2015, there have been several advances in the technique, making it increasingly less invasive and requiring partial laminectomy in only a small number of cases (26). Recently, Deschamps and colleagues reported the results of ECT treatment in 40 patients with MESCC: ECT significantly improved pain and neurological symptoms in 80 and 55% of the patients, respectively. Complication was reported to be minor overall, with 7.5% rate of severe complications. Thus, the EP technique proved to be safe and effective for the treatment of spinal metastases. However, specific designs and geometries of the electrode needle aim to simplify the procedure by reducing the number of needles, shortening insertion times, minimizing invasiveness, improving accuracy. Our study evaluated the effects of the EP treatment in healthy vertebrae and surrounding structures of clinical relevance in an ovine model, with the new coaxial bipolar electrode used either individually or in pairs.

Results showed that in the sheep vertebrae, the ablation induced by EP was easily discernible 7 days following surgery and electric field application both when 1 and 2 bipolar electrodes were used. Upon histological examination, an array of significant findings emerged, including the detachment of osteoblasts from trabeculae, transient inhibition of bone apposition, the presence of pyknotic osteocytes and vacated lacunae. Remarkably, the application of electrodes without the concomitant application of an electric field yielded no discernible impact on the viability of bone tissue or mineral apposition, highlighting the specificity and efficacy of the EP technique in targeted tissue ablation. These results were further confirmed by the presence of tetracycline fluorescence in the sheep vertebrae treated with electrodes without the application of an electric field, and by the absence of tetracycline fluorescence in the sheep vertebrae treated with EP, indicating the complete absence of bone tissue growth in the ablated area. In terms of histomorphometry, no ablated bone area was present in the control vertebrae, confirming that the surgical procedure did not affect cell viability. Whereas differences in the ablated area were observed between the L2 vertebral body, where the electric field was applied with one bipolar electrode, and the L3 and L4 vertebrae, where the electric field was applied by 2 coupled bipolar electrodes. Indeed, when the electric field is applied with one electrode, an ablation diameter of 11.22 ± 0.97 mm (ablation area: 99.56 ± 18.00 mm^2^) around the electrode was observed, whereas when the field was applied with 2 bipolar electrodes, the ablation diameter was significantly larger, 17.20 ± 3.04 mm (ablation area: 238.97 ± 81.44 mm^2^). This aspect holds critical importance in clinical applications, particularly in the context of treating spinal metastases.

These results are also supported by the numerical evaluation of treatments applied to vertebrae L2 and L4, where simulations were conducted using coaxial bipolar electrodes individually or in pairs, respectively. Numerical simulations showed the distribution of the AD in the vertebral body and in the nearby spinal cord. Based on the AD threshold for tissue ablation of 3,500 J/kg (35), the estimated ablated volume within L2 and L4 vertebral bodies is 0.43 cm^3^ and 3.45 cm^3^, respectively.

Our results underscore the need to precisely ablate both smaller and larger areas, representing a significant step forward in surgical practice. It would allow for increasingly targeted treatments, optimizing the effectiveness of surgical intervention and minimizing the risk of complications. This would result in better overall outcomes for patients, enhancing their prognosis and post-operative quality of life. Additionally, when treating spinal metastases, it is crucial to consider the potential damage to the spinal cord and nerves, particularly because both structures are frequently located within the tumor margin. Our study demonstrated no deficits attributable to the electroporation in spinal nerves and the spinal cord when bipolar electrodes were used either individually or in pairs, further highlighting the feasibility and safety of the procedure in the spine.

To further validate the safety of the EP procedure for the treatment of spinal metastases, the temperature variation (ΔT) in spinal cord was estimated. The predicted temperature increase in the spinal cord at the level of the treated vertebra was below 0.01 °C for L2 and below 0.05 °C for L4, reaching a maximum increase of 0.025 °C and 0.25 °C in L2 and L4, respectively.

An early investigation conducted in dogs demonstrated that the maximum tolerable temperature of the spinal cord should not exceed 42 °C (43). Dewhirst et al. (44) further reported that the thresholds for thermal damage to spinal cord and brain are consistent across species, indicating that thermal damage threshold for the mouse spinal cord is 100 min at 42 °C. More recently, Konno et al. applied controlled temperature increases by means of a heat-generating probe to the cauda equina nerve roots in pigs, demonstrating that exposure to 40 °C for 5 min did not cause any changes in nerve root function (45). Based on these findings, together with the estimated temperature increase and on our histological analysis of both spinal cord and nerves, we can reasonably exclude the possibility of EP-induced thermal damage. These conclusions are consistent with our previous preclinical study (25) and the clinical outcomes where overall minor complications have been reported, with 7.5% rate of severe complications (26–27, 46). Moreover, a study investigating direct electroporation-mediated ablation of the spinal canal in pigs (47) illustrated that the procedure can be performed directly adjacent to the spinal cord with minimal adverse effects, likely attributable to the anatomical structure of the spinal canal. The presence of epidural fat surrounding the spinal cord serves as an electrically protective layer. Given the low electrical conductivity of adipose tissue, the primary voltage drops, and consequent electric field strength, occur within the epidural space rather than within the spinal cord (48).

Based on our study, we have demonstrated that EP-mediated ablation with coaxial bipolar electrode within the vertebral body, utilizing a transpedicular approach, represents a safe and minimally invasive treatment option for spinal tumors and metastases, regardless of lesion size, while preserving critical neural structures.

## Conclusion

This preclinical study provides robust evidence supporting the safety and efficacy of electroporation-based treatments for spinal metastases. The use of coaxial bipolar electrodes, either individually or in pairs, enabled precise and controlled ablation of bone tissue without compromising adjacent neurological structures. Histological analyses and thermal modeling confirmed the absence of electroporation-induced thermal damage, while numerical simulations highlighted the critical role of electrode configuration in determining ablation volume. These findings underscore the potential of electroporation as a minimally invasive, targeted therapeutic approach for spinal lesions. Furthermore, the technique’s ability to preserve spinal cord and nerve integrity reinforces its clinical feasibility and paves the way for its integration into the treatment algorithm for spinal metastases. Future clinical studies are warranted to validate these results and optimize treatment protocols for broader clinical application.

## Data Availability

The raw data supporting the conclusions of this article will be made available by the authors, without undue reservation.

## References

[1] Maccauro G Spinelli MS Mauro S Perisano C Graci C Rosa MA. Physiopathology of spine metastasis. Int J Surg Oncol. (2011) 2011:107969. doi: 10.1155/2011/107969, PMID: 22312491 PMC3265280

[2] Ribi K Thürlimann B Schär C Dietrich D Cathomas R Zürrer-Härdi U. Quality of life and pain in patients with metastatic bone disease from solid tumors treated with bone-targeted agents– a real-world cross-sectional study from Switzerland (SAKK 95/16). BMC Cancer. (2021) 21:182. doi: 10.1186/s12885-021-07903-8, PMID: 33607966 PMC7893880

[3] Kimura T. Multidisciplinary approach for bone metastasis: a review. Cancers (Basel). (2018) 10:156. doi: 10.3390/cancers10060156, PMID: 29795015 PMC6025143

[4] Orthobullets. (2025). Available at: https://www.orthobullets.com/pathology/2009/metastatic-disease-of-spine.

[5] Galbusera F Bassani T. The spine: a strong, stable, and flexible structure with biomimetics potential. Biomimetics (Basel). (2019) 4:60. doi: 10.3390/biomimetics4030060, PMID: 31480241 PMC6784295

[6] Seyfried TN Huysentruyt LC. On the origin of cancer metastasis. Crit Rev Oncog. (2013) 18:43–73. doi: 10.1615/critrevoncog.v18.i1-2.40, PMID: 23237552 PMC3597235

[7] Conti A Acker G Kluge A Loebel F Kreimeier A Budach V. Decision making in patients with metastatic spine. The role of minimally invasive treatment modalities. Front Oncol. (2019) 9:915. doi: 10.3389/fonc.2019.00915, PMID: 31608228 PMC6761912

[8] Ahangar P Aziz M Rosenzweig DH Weber MH. Advances in personalized treatment of metastatic spine disease. Ann Transl Med. (2019) 7:223. doi: 10.21037/atm.2019.04.41, PMID: 31297388 PMC6595197

[9] Igoumenou VG Mavrogenis AF Angelini A Baracco R Benzakour A Benzakour T. Complications of spine surgery for metastasis. Eur J Orthop Surg Traumatol. (2020) 30:37–56. doi: 10.1007/s00590-019-02541-0, PMID: 31473821

[10] Kwak K Yu B Lewandowski RJ Kim DH. Recent progress in cryoablation cancer therapy and nanoparticles mediated cryoablation. Theranostics. (2022) 12:2175–204. doi: 10.7150/thno.67530, PMID: 35265206 PMC8899563

[11] Batista Napotnik T Polajžer T Miklavčič D. Cell death due to electroporation - a review. Bioelectrochemistry. (2021) 141:107871. doi: 10.1016/j.bioelechem.2021.107871, PMID: 34147013

[12] Gehl J. Drug and gene Electrotransfer in Cancer therapy In: Li XQ Donnelly D Jensen T, editors. Somatic genome manipulation. New York, NY: Springer (2015)

[13] Rubinsky B Onik G Mikus P. Irreversible electroporation: a new ablation modality--clinical implications. Technol Cancer Res Treat. (2007) 6:37–48. doi: 10.1177/153303460700600106, PMID: 17241099

[14] Cadossi R Massari L Racine-Avila J Aaron RK. Pulsed electromagnetic field stimulation of bone healing and joint preservation: cellular mechanisms of skeletal response. J Am Acad Orthop Surg Glob Res Rev. (2020) 4:e1900155. doi: 10.5435/JAAOSGlobal-D-19-00155, PMID: 33970582 PMC7434032

[15] Marty M Sersa G Garbay JR Gehl J Collins CG Snoj M. Electrochemotherapy – an easy, highly effective and safe treatment of cutaneous and subcutaneous metastases: results of ESOPE (European standard operating procedures of electrochemotherapy). Eur J Cancer Suppl. (2006) 4:3–13. doi: 10.1016/j.ejcsup.2006.08.002

[16] Quaglino P Mortera C Osella-Abate S Barberis M Illengo M Rissone M. Electrochemotherapy with intravenous bleomycin in the local treatment of skin melanoma metastases. Ann Surg Oncol. (2008) 15:2215–22. doi: 10.1245/s10434-008-9976-0, PMID: 18498012

[17] Campana LG Mocellin S Basso M Puccetti O De Salvo GL Chiarion-Sileni V. Bleomycin-based electrochemotherapy: clinical outcome from a single institution's experience with 52 patients. Ann Surg Oncol. (2009) 16:191–9. doi: 10.1245/s10434-008-0204-8, PMID: 18987914

[18] Ringe KI Panzica M von Falck C. Thermoablation of bone tumors. Rofo. (2016) 188:539–50. doi: 10.1055/s-0042-100477, PMID: 26981915

[19] Probst S Bjartell A Anand A Skamene T Ferrario C. Interval changes in PSMA PET/CT during Radium-223 therapy for metastatic bone disease from castration-resistant prostate Cancer. Nucl Med Mol Imaging. (2022) 56:188–95. doi: 10.1007/s13139-022-00754-6, PMID: 35846415 PMC9276891

[20] Errani C Bazzocchi A Spinnato P Facchini G Campanacci L Rossi G. What’s new in management of bone metastases? Eur J Orthop Surg Traumatol. (2019) 29:1367–75. doi: 10.1007/s00590-019-02446-y, PMID: 31089821

[21] Campanacci L Cevolani L De Terlizzi F Saenz L Alì N Bianchi G. Electrochemotherapy Is Effective in the Treatment of Bone Metastases. Curr Oncol. (2022) 29:1672–82. doi: 10.3390/curroncol29030139, PMID: 35323339 PMC8947745

[22] Campanacci L Bianchi G Cevolani L Errani C Ciani G Facchini G. Operating procedures for electrochemotherapy in bone metastases: Results from a multicenter prospective study on 102 patients. Eur J Surg Oncol. (2021) 47:2609–17. doi: 10.1016/j.ejso.2021.05.004, PMID: 34083080

[23] Bianchi G Campanacci L Ronchetti M Donati D. Electrochemotherapy in the Treatment of Bone Metastases: A Phase II Trial. World J Surg. (2016) 40:3088–94. doi: 10.1007/s00268-016-3627-6, PMID: 27443372 PMC5104781

[24] Malyško-Ptašinskė V Staigvila G Novickij V. Invasive and non-invasive electrodes for successful drug and gene delivery in electroporation-based treatments. Front Bioeng Biotechnol. (2023) 10:1094968. doi: 10.3389/fbioe.2022.1094968, PMID: 36727038 PMC9885012

[25] Tschon M Salamanna F Ronchetti M Cavani F Gasbarrini A Boriani S. Feasibility of electroporation in bone and in the surrounding clinically relevant structures: a preclinical investigation. Technol Cancer Res Treat. (2016) 15:737–48. doi: 10.1177/1533034615604454, PMID: 26351303

[26] Gasbarrini A Campos WK Campanacci L Boriani S. Electrochemotherapy to metastatic spinal melanoma: a novel treatment of spinal metastasis? Spine (Phila Pa 1976). (2015) 40:E1340–6. doi: 10.1097/BRS.0000000000001125 26274530

[27] Deschamps F Tselikas L Yevich S Bonnet B Roux C Kobe A. Electrochemotherapy in radiotherapy-resistant epidural spinal cord compression in metastatic cancer patients. Eur J Cancer. (2023) 186:62–8. doi: 10.1016/j.ejca.2023.03.012, PMID: 37030078

[28] Cornelis FH Ben Ammar M Nouri-Neuville M Matton L Benderra MA Gligorov J. Percutaneous image-guided electrochemotherapy of spine metastases: Initial Experience. Cardiovasc Intervent Radiol. (2019) 42:1806–9. doi: 10.1007/s00270-019-02316-4, PMID: 31440783

[29] Merola G Fusco R Di Bernardo E D’Alessio V Izzo F Granata V. Design and characterization of a minimally invasive bipolar electrode for electroporation. Biology. (2020) 9:303. doi: 10.3390/biology9090303, PMID: 32967343 PMC7563710

[30] Caffrey JM Thomas PK Appt SE Burkart HB Weaver CM Kleinberger M. Contrast enhanced computed tomography of small ruminants: caprine and ovine. PLoS One. (2023) 18:e0287529. doi: 10.1371/journal.pone.0287529, PMID: 38127918 PMC10735035

[31] Cindrič H Miklavčič D Cornelis FH Kos B. Optimization of transpedicular electrode insertion for electroporation-based treatments of vertebral Tumors. Cancers (Basel). (2022) 14:5412. doi: 10.3390/cancers14215412, PMID: 36358829 PMC9657605

[32] Cindrič H Kos B Tedesco G Cadossi M Gasbarrini A Miklavčič D. Electrochemotherapy of spinal metastases using transpedicular approach-a numerical feasibility study. Technol Cancer Res Treat. (2018) 17:1533034618770253. doi: 10.1177/1533034618770253, PMID: 29759043 PMC5956634

[33] Hasgall PA Neufeld E Gosselin MC Klingenböck A Kuster N. IT’IS database for thermal and electromagnetic parameters of biological tissues (2022). doi: 10.13099/VIP21000-04-1,

[34] Ibey BL Xiao S Schoenbach KH Murphy MR Pakhomov AG. Plasma membrane permeabilization by 60- and 600-ns electric pulses is determined by the absorbed dose. Bioelectromagnetics. (2009) 30:92–9. doi: 10.1002/bem.20451, PMID: 18839412 PMC2632729

[35] Fini M Tschon M Ronchetti M Cavani F Bianchi G Mercuri M. Ablation of bone cells by electroporation. J Bone Joint Surg Br. (2010) 92-B:1614–20. doi: 10.1302/0301-620X.92B11.24664, PMID: 21037363

[36] Becker SM. (2015). Chapter 4 - analytical bioheat transfer: Solution development of the Pennes’ model. In, S. M. Becker, A. V. Kuznetsov, (eds.) Heat transfer and fluid flow in biological processes. Boston: Academic Press, 77–124. doi: 10.1016/B978-0-12-408077-5.00004-3

[37] Galluzzi L Vitale I Aaronson SA Abrams JM Adam D Agostinis P. Molecular mechanisms of cell death: recommendations of the nomenclature committee on cell death 2018. Cell Death Differ. (2018) 25:486–541. doi: 10.1038/s41418-017-0012-4, PMID: 29362479 PMC5864239

[38] Coleman William B. Tsongalis Gregory J. Chapter 1 - molecular mechanisms of cell death. Molecular pathology (Second Edition), (2018), pp. 1–24

[39] Tang D Kang R Berghe TV Vandenabeele P Kroemer G. The molecular machinery of regulated cell death. Cell Res. (2019) 29:347–64. doi: 10.1038/s41422-019-0164-5, PMID: 30948788 PMC6796845

[40] Thomson KR Cheung W Ellis SJ Federman D Kavnoudias H Loader-Oliver D. Investigation of the safety of irreversible electroporation in humans. J Vasc Interv Radiol. (2011) 22:611–21. doi: 10.1016/j.jvir.2010.12.014, PMID: 21439847

[41] Scheffer HJ Nielsen K de Jong MC van Tilborg AA Vieveen JM Bouwman AR. Irreversible electroporation for nonthermal tumor ablation in the clinical setting: a systematic review of safety and efficacy. J Vasc Interv Radiol. (2014) 25:997–1011. doi: 10.1016/j.jvir.2014.01.028, PMID: 24656178

[42] Narayanan G Bhatia S Echenique A Suthar R Barbery K Yrizarry J. Vessel patency post irreversible electroporation. Cardiovasc Intervent Radiol. (2014) 37:1523–9. doi: 10.1007/s00270-014-0988-9, PMID: 25212418

[43] Uchiyama S Yashiro K Takahashi H Homma T. An experimental study of spinal cord evoked potentials and histologic changes following spinal cord heating. Spine (Phila Pa 1976). (1989) 14:1215–9. doi: 10.1097/00007632-198911000-00014, PMID: 2603055

[44] Dewhirst MW Viglianti BL Lora-Michiels M Hoopes PJ Hanson M. Thermal dose requirement for tissue effect: experimental and clinical findings. Proc SPIE Int Soc Opt Eng. (2003) 4954:37. doi: 10.1117/12.476637, PMID: 25301982 PMC4188373

[45] Konno S Olmarker K Byröd G Nordborg C Strömqvist B Rydevik B. The European spine society AcroMed prize 1994. Acute thermal nerve root injury. Eur Spine J. (1994) 3:299–302. doi: 10.1007/BF02200140, PMID: 7866856

[46] Cornelis FH Bilhim T Hacking N Sapoval M Tapping CR Carnevale FC. CIRSE standards of practice on prostatic artery embolisation. Cardiovasc Intervent Radiol. (2020) 43:176–85. doi: 10.1007/s00270-019-02379-3, PMID: 31792588

[47] Dunki-Jacobs EM Philips P Martin RC 2nd. Evaluation of thermal injury to liver, pancreas and kidney during irreversible electroporation in an in vivo experimental model. Br J Surg. (2014) 101:1113–21. doi: 10.1002/bjs.9536, PMID: 24961953

[48] Tam AL Figueira TA Gagea M Ensor JE Dixon K McWatters A. Irreversible electroporation in the epidural space of the porcine spine: effects on adjacent structures. Radiology. (2016) 281:763–71. doi: 10.1148/radiol.2016152688, PMID: 27266723 PMC5952366

