# Supplementary material for: Application of a new coaxial bipolar electrode for the treatment of vertebral metastases: a pilot study in an ovine model
Source: Front Med (Lausanne). 2025 Sep 24;12:1627296. doi: 10.3389/fmed.2025.1627296 (PMC12504192; doi:10.3389/fmed.2025.1627296)
Supplement: Supplementary file 1 [file Data_Sheet_1.docx]

**SUPPLEMENTARY MATERIAL**

***Section I - Equations underlying the simulative models***

The equations solved in the “Electric currents” physics interface implemented in COMSOL are based on Maxwell’s equations for electromagnetism in the time domain and are described as follows:

(1) $\nabla\cdot J=Q_{j,v}$

(2) $J= \sigma E+ \frac{dD}{dt}+ J_{e}$

(3) $E= -\nabla V$

Where **J** is the current density vector [A/m^2^], Q_j,v_ is the current source [A/m^3^], **E** is the electric field vector [V/m], σ is the medium electrical conductivity [S/m], which in turn will depend on the absolute value of the applied E-field, as explained in Cindrič et al., 2022. d**D**/dt is the time derivate of the electric displacement field vector **D** [C/m^2^], **J_e_** is the externally applied current density vector [A/m^2^] and V is the scalar electric potential [V].

The solver consider the scalar electric potential V applied to the electrodes as the dependent variable in order to solve the equations in each tetrahedron of the mesh. The equations consider the electrical conductivity and relative permittivity values of each tetrahedron in the model to solve the characteristic equations of the medium.

Moreover, the boundary conditions ensure continuity between the edges of the different tetrahedrons:

(4) $J_{1}n_{1}=J_{2}n_{2}$

Where **J_1_,J_2_** are the current densities of the adjacent elements 1 and 2, respectively, and **n_1_, n_2_** are the vectors normal to each surface of the tetrahedrons.

Full reference: Cindrič H.; Miklavčič, D.; Cornelis, F.H.; Kos, B. Optimization of Transpedicular Electrode Insertion for Electroporation-Based Treatments of Vertebral Tumors. *Cancers* **2022**, *14*, 5412. https://doi.org/10.3390/cancers14215412

***Section II – Estimation of Temperature Increase Using the Pennes’ Bioheat Equation***

To estimate the temperature increase induced by electromagnetic exposure, we applied the steady-state form of the Pennes’ bioheat equation (5):

$\rho c\frac{\partial T}{\partial t}=\nabla\cdot\left( k\nabla T \right)+Q_{b}+Q_{met}+Q_{Joule}$ (5)

where: ρ is the tissue density (kg/m³), c is the specific heat capacity of the tissue (J/kg·K), T is the temperature (K), k is the thermal conductivity (W/m·K), Q_b_​ is the heat exchange with blood (W/m³), typically representing perfusion effects, Q_met_ is the metabolic heat generation rate (W/m³) and Q_Joule_​ is the Joule heating source term representing power deposited by electromagnetic exposure (W/m³). This term is defined by (6):

$Q_{Joule}=\rho\cdot SAR$ (6)

where SAR is the specific absorption rate (W/kg), defined as the electromagnetic power absorbed per unit mass.

However, since we aimed to obtain a first-order estimation of the temperature variation, we simplified the equation by neglecting thermal conduction, perfusion, and metabolic heat generation rate. Thus, assuming a constant SAR during the exposure duration leads to the simplified form of Eq. (5), where tₑₓₚ is the exposure duration:

$\Delta T\approx\frac{SAR}{c}\cdot t_{exp}$ (7)

Tissue-specific parameters were taken from IT’IS Foundation database (32). This approach provides a conservative estimation of temperature elevation, useful for preliminary safety evaluation in the absence of full thermodynamic modeling.

***Supplementary Table 1*** ***– Dielectric properties of modelled tissues and electrodes***

| **Tissue** | **Initial Conductivity (σ_0_ [S/m])** | **Factor of increase (f)** |
| --- | --- | --- |
| Cortical Bone | 0.02 | 3.0 |
| Cancellous Bone | 0.07 | 2.9 |
| Spinal Cord | 0.23 | 3.0 |
| Cerebrospinal Fluid | 1.50 | 1.0 |
| Intervertebral Disc | 1.00 | 3.0 |
| Electrodes – active part (Steel AISI 4340) | 4 · 10^6^ | - |
| Electrodes – separation part (Polyimide) | 1 · 10^-16^ | - |

***Supplementary Figure 1 – Bipolar coaxial electrode***


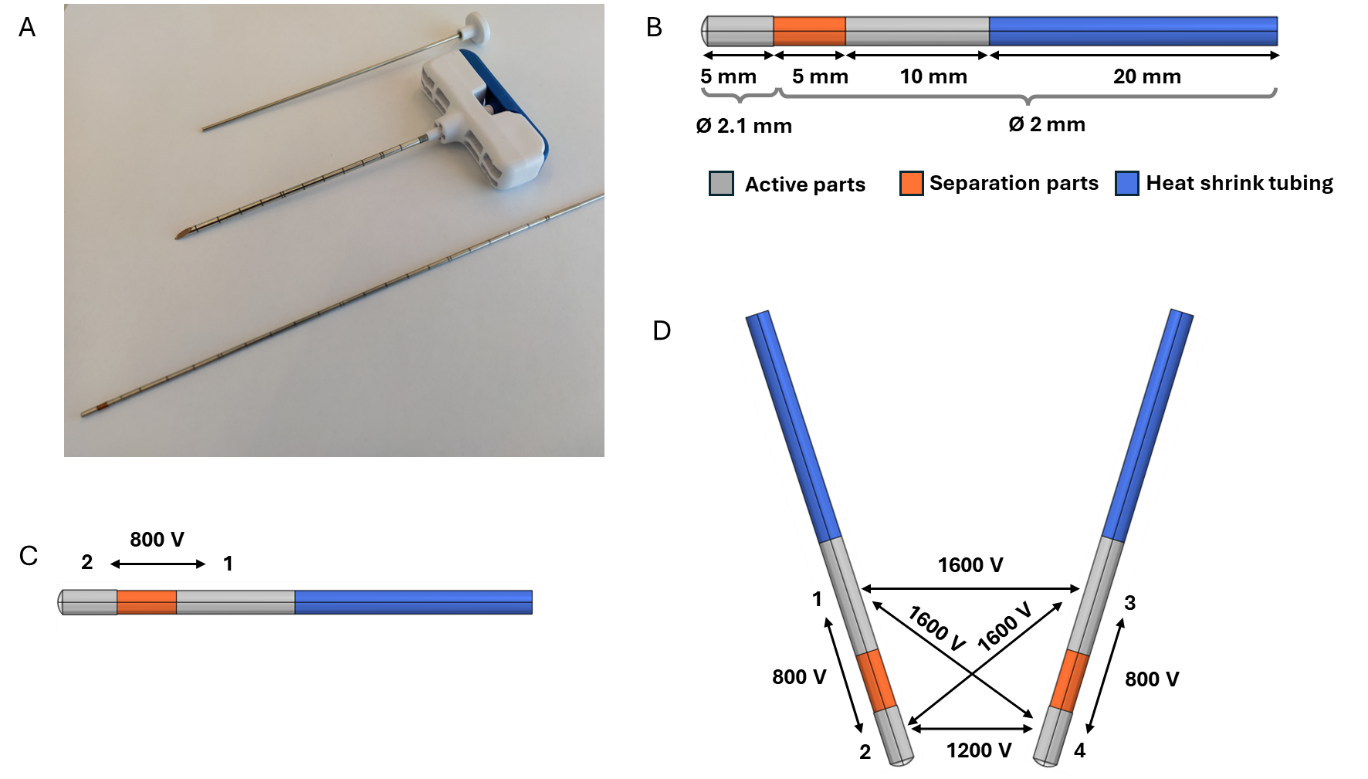


**Supplementary Figure 1**. A) Image showing the bipolar coaxial needle and the trocar for percutaneous access. B) Electrode geometry. C) Schematic representation of EP-ablation treatment for single bipolar electrode. D) Schematic representation of EP-ablation treatment for bipolar electrodes used in pairs.
